# Efficient production of a functional G protein-coupled receptor in *E. coli* for structural studies

**DOI:** 10.1007/s10858-020-00354-6

**Published:** 2021-01-27

**Authors:** Layara Akemi Abiko, Marco Rogowski, Antoine Gautier, Gebhard Schertler, Stephan Grzesiek

**Affiliations:** 1 Focal Area Structural Biology and Biophysics, Biozentrum, University of Basel, 4056 Basel, Switzerland; 2 Paul Scherrer Institute, 5232 Villigen, Switzerland

**Keywords:** G protein-coupled receptor, Heterologous *E. coli* expression, Cloning, Structural biology, Solution NMR

## Abstract

G protein-coupled receptors (GPCRs) are transmembrane signal transducers which regulate many key physiological process. Since their discovery, their analysis has been limited by difficulties in obtaining sufficient amounts of the receptors in high-quality, functional form from heterologous expression hosts. Albeit highly attractive because of its simplicity and the ease of isotope labeling for NMR studies, heterologous expression of functional GPCRs in *E. coli* has proven particularly challenging due to the absence of the more evolved protein expression and folding machinery of higher eukaryotic hosts. Here we first give an overview on the previous strategies for GPCR *E. coli* expression and then describe the development of an optimized robust protocol for the *E. coli* expression and purification of two mutants of the turkey β_1_-adrenergic receptor (β_1_AR) uniformly or selectively labeled in ^15^N or ^2^H,^15^N. These mutants had been previously optimized for thermal stability using insect cell expression and used successfully in crystallographic and NMR studies. The same sequences were then used for *E. coli* expression. Optimization of *E. coli* expression was achieved by a quantitative analysis of losses of receptor material at each step of the solubilization and purification procedure. Final yields are 0.2–0.3 mg receptor per liter culture. Whereas both expressed mutants are well folded and competent for orthosteric ligand binding, the less stable YY-β_1_AR mutant also comprises the two native tyrosines Y^5.58^ and Y^7.53^, which enable G protein binding. High-quality ^1^H-^15^N TROSY spectra were obtained for *E. coli*-expressed YY-β_1_AR in three different functional states (antagonist, agonist, and agonist + G protein-mimicking nanobody-bound), which are identical to spectra obtained of the same forms of the receptor expressed in insect cells. NdeI and AgeI restriction sites introduced into the expression plasmid allow for the easy replacement of the receptor gene by other GPCR genes of interest, and the provided quantitative workflow analysis may guide the respective adaptation of the purification protocol.

**Supplementary Information:**

The online version of this article (10.1007/s10858-020-00354-6) contains supplementary material, which is available to authorized users.

## Introduction

The more than 800 G protein-coupled receptors (GPCRs) constitute the most abundant class of membrane proteins in the human proteome (Alexander et al. 2017) and also the most important class of human drug targets, with more than 30% of all marketed drugs directed against GPCRs (Hauser et al. 2017). Since their discovery, the production of properly folded, active GPCRs has been the major bottleneck for biochemical, biophysical and structural studies (Tate 2001; Lundstrom et al. 2006; McCusker et al. 2007; Casiraghi et al. 2018). Despite many advances in protein engineering, cloning, expression, and purification technologies, which have led to the experimental elucidation of currently more than 80 distinct GPCR structures, GPCR production in sufficient quantity and quality is still a serious challenge (Wiseman et al. 2020), with low expression levels, low stability, misfolding, aggregation, incorrect disulfide bond formation, and proteolytic degradation being the main impeding factors.

GPCRs have been expressed in many different hosts comprising mammalian cells, insect cells, yeast and *E. coli* with each system presenting particular advantages and disadvantages [for recent review see (Wiseman et al. 2020)]. The most successful system are baculovirus-infected insect cells, from which about 70% of all solved GPCR structures have been derived (Milić and Veprintsev 2015; Saarenpää et al. 2015; Franke et al. 2018) This success is based on their relative ease of maintenance as compared to mammalian cells, while still harboring the evolved protein translational, folding, membrane insertion, and posttranslational modification mechanisms of higher eukaryotes, which are less developed or absent in lower cellular systems such as yeast and *E. coli.* Disadvantages of the insect cell system comprise the tedious generation of baculoviruses, lengthy culture processes, and, with respect to high-resolution heteronuclear NMR, the requirement to supply isotope-labeled amino acids to the medium, since insect cells do not produce amino acids from simple precursors like glucose and ammonium. The latter disadvantage can be alleviated to some extent in a cost-effective manner by feeding isotope-labeled yeast or algal extracts to the insect cells (Franke et al. 2018).

In contrast, expression in *E. coli* offers several particular advantages, such as inexpensive (isotope-labeled) growth media, easy genetic modification, growth to high cell densities, and simple and fast scale-up processes. These features have made *E. coli* the most used expression system for soluble proteins. However, heterologous expression of eukaryotic membrane proteins is considerably more difficult in *E. coli* for numerous reasons, comprising the lack of the endoplasmic reticulum and Golgi apparatus quality control and the more evolved post-translational modification (PTM) and membrane insertion machineries present in eukaryotes as well as inherent toxicity. Nevertheless, many examples exist of the successful *E. coli* expression of important eukaryotic membrane proteins, including GPCRs. In order to overcome the described difficulties, almost always empirical optimization of diverse parameters such as expression constructs, *E. coli* strains, induction, temperature, etc. is required. Without doubt, protein engineering towards higher stability has been the most import step to the success of GPCR structural studies both in eukaryotic and non-eukaryotic expression systems. Such stabilizing sequence modifications comprise loop deletions, protein insertions (T4 lysozyme, rubredoxin, etc.), and extensive point mutations (Cherezov et al. 2007; Rosenbaum et al. 2007; Warne et al. 2008; Sarkar et al. 2008; Shibata et al. 2009; Miller and Tate 2011). An ingenious method for directed evolution of GPCRs in *E. coli* towards high expression and stability has been developed by the Plückthun group (Sarkar et al. 2008; Dodevski and Plückthun 2011; Schlinkmann et al. 2012) using iterative rounds of gene randomization and selection based on fluorescence-labeled ligand binding.

Although the work described here uses *E.coli* in-cell expression of GPCRs, we briefly want to mention that also cell-free synthesis is possible in *E.coli* lysates (Henrich et al. 2015). While the latter method has been successful for the production of certain ligand-binding competent GPCRs (Klammt et al. 2007; Kögler et al. 2019), a recent NMR characterization of the cell-free expressed neurotensin receptor 1 indicated a lack of tertiary structure and that further improvements to the method were necessary (Shilling et al. 2017).

In the following, we first give an overview on previous strategies to produce GPCRs for structural studies from in-cell *E. coli* expression and then further develop these into a robust, quantitative protocol for the *E. coli* production of two turkey β_1_-adrenergic receptor (β_1_AR) mutants, which had previously been optimized for thermal stability in insect cells and expressed in these eukaryotic cells leading to successful crystallization (Warne et al. 2008) and detailed NMR studies (Isogai et al. 2016; Abiko et al. 2019; Grahl et al. 2020). The first ultrastable β_1_AR mutant (TS-β_1_AR, melting temperature T_m_ = 59 °C), harboring 12 point mutations and truncations at the N- and C-termini, is competent for orthosteric ligand binding, but deficient in G protein activation (Isogai et al. 2016). The second TS-β_1_AR_A227Y/L343Y_ double mutant (named YY-β_1_AR in the following) recovers G protein activation by reintroducing the conserved tyrosines Y^5.58^ and Y^7.53^ [the superscript corresponds to Ballesteros-Weinstein numbering (Ballesteros and Weinstein 1995)] in TM5 and TM7 at the expense of stability (T_m_ = 48 °C) (Isogai et al. 2016). Both mutants do not carry PTMs such as palmitoylation, which were not necessary for successful structural studies. The 2D ^1^H-^15^N NMR spectra of different ligand-bound forms of the *E. coli*-expressed β_1_AR mutants including a ternary complex of YY-β_1_AR with the agonist isoprenaline and the G protein mimicking nanobody Nb80 (Rasmussen et al. 2011) are identical to spectra obtained from insect-cell expressed receptors. The described protocol for *E. coli* β_1_AR expression and purification should be applicable to other GPCRs of sufficient stability. Such an adaptation is aided by the quantification of receptor amounts at each step of the purification protocol and by the introduction of restriction sites into the expression plasmid, which allow an easy replacement of β_1_AR by other GPCR genes of interest.

## Results and discussion

### Overview of previous strategies for GPCR expression and purification using *E. coli*

**Fig. 1:**
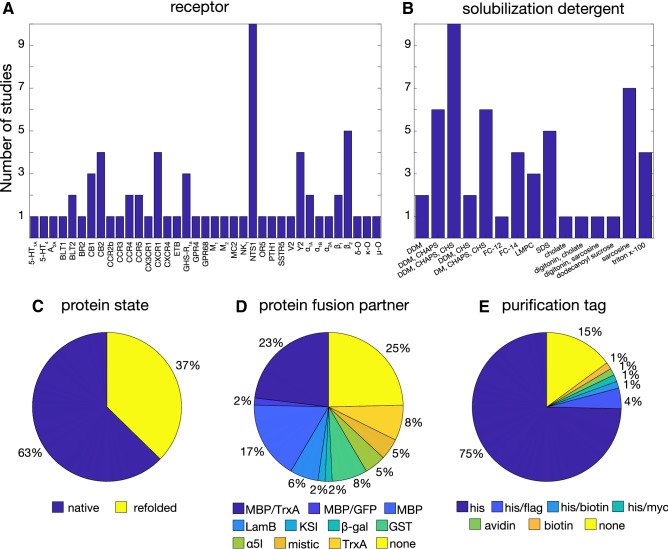
Overview of the published studies on GPCR expression in *E. coli* and statistical analysis of the used expression and purification strategies: **a** types of receptors, **b** detergents, **c** expression strategy, **d** protein fusion partners, and **e** purification tags. Details of the used data are provided in Supplementary Table S1

To learn from past experience, we reviewed 50 of the previous studies on GPCR expression in *E. coli* (Supplementary Table S1). They cover 37 distinct receptors, with the neurotensin receptor (NTS1) being the most studied (Fig. 1a). Of the analyzed protocols, 37% used expression in inclusion bodies and refolding, whereas 63% aimed at the insertion of the properly folded receptor into the *E. coli* inner membrane (Fig. 1c).

Expression in inclusion bodies usually generates high amounts of material, but refolding can be very challenging for GPCRs in particular with regard to finding the proper membrane mimetics and the correct formation of disulfide bonds. Nevertheless, the CXCR1, Y2, and GRH-R1a receptors have been obtained from *E. coli* inclusion bodies and refolded into phospholipid bilayers for successful structure determination, modeling or dynamics measurements using solid-state NMR data (Park et al. 2012; Schmidt et al. 2014; Schrottke et al. 2017; Bender et al. 2019).

Expression directed to the membrane has been used for many different GPCRs (Bertin et al. 1992; Tucker and Grisshammer 1996; Stanasila et al. 1999; Furukawa and Haga 2000; Hampe et al. 2000; Weiß and Grisshammer 2002; Yeliseev et al. 2005; Grisshammer et al. 2005; Dodevski and Plückthun 2011) and led to successful structural studies of NTS1 (Egloff et al. 2014; Nasr et al. 2017), α_1A_ (Wu et al. 2020), and α_1B_ (Schuster et al. 2020). In almost all cases, membrane insertion has been achieved with an expression construct originally described for NTS1 (Tucker and Grisshammer 1996). In this construct (pRG/III-hs-MBP), the receptor is fused at the N-terminus to the periplasmic maltose-binding protein (MBP) together with its signaling peptide, as well as at the C-terminus to the cytoplasmic thioredoxin A (TrxA) followed by a histidine tag. The N-terminal MBP fusion directs the first GPCR helix towards the periplasm, thereby inducing the correct orientation of the following helices, such that the outside of the folded receptor points to the periplasm and the inside to the cytoplasm. MBP is by far the most used fusion protein (42%, Fig. 1d) for GPCR *E. coli* expression. The C-terminal TrxA fusion is not important for the proper membrane insertion, but increased the expression levels of many receptors (NTS1, CB2, NK1, α_1A_, α_2A_, CCR3, CCR5, CX3CR1, CXCR4), which appears due a stabilizing effect by this soluble, compact protein (Tucker and Grisshammer 1996; Yeliseev et al. 2005; Ren et al. 2009; Dodevski and Plückthun 2011). Other stable, cytosolic proteins such as glutathione S-transferase (GST) have also been found to increase GPCR expression yield and stability in inclusion bodies (Kiefer et al. 1996; Park et al. 2012) (Fig. 1d).

Of note, also low temperature (16–25 °C) and low inducer concentrations (0.5–0.1 mM IPTG) have been used for the majority of natively expressed GPCRs in *E. coli* to slow the transcription rate and ensure correct translocation to the membrane without aggregation (Supplementary Table S1).

Once the problems of heterologous expression and membrane insertion are overcome, the next challenging step is the extraction from the host membrane. For GPCRs, the most common extraction detergents are the maltosides DDM and DM mixed with cholesteryl hemisuccinate (CHS), or zwitterionic surfactants such as CHAPS (Fig. 1b). It should be noted that although alkylphosphocholine (FC) detergents have been widely used for solution NMR studies of membrane proteins, there are now many examples showing their destabilizing and inactivating effects in particular for α-helical proteins (Chipot et al. 2018).

For immunodetection and downstream purification of the detergent-solubilized receptors histidine tags fused to the C-terminal region are used in most (81%) cases (Fig. 1e). In a direct comparison, a His_10_ tag proved more efficient for nickel affinity purification of NTS1 than a His_6_ tag (Grisshammer and Tucker 1997).

In the following, we have translated and optimized these strategies to the high-yield *E. coli* expression and purification of the two TS-β_1_AR and YY-β_1_AR mutants of the turkey β_1_AR, which we had previously used in isotope-labeled form from insect cells for NMR dynamical studies (Isogai et al. 2016; Abiko et al. 2019; Grahl et al. 2020).

### Optimization of the expression vector

**Fig. 2:**
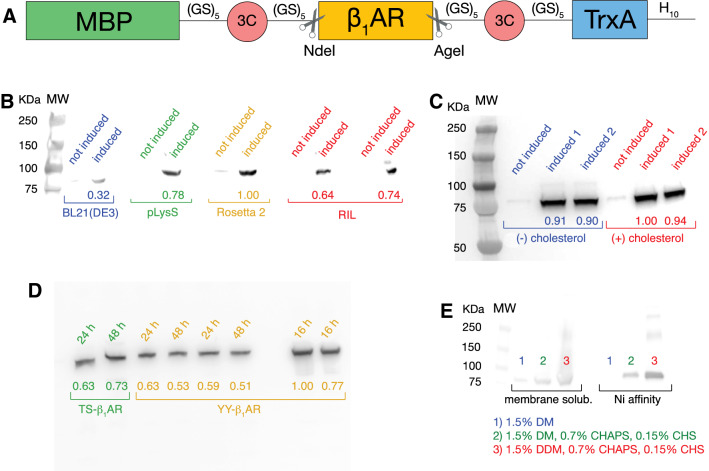
Fusion protein construct for β_1_AR expression in *E. coli* and tests for expression and solubilization optimization. **a** Fusion protein construct for *E. coli* expression based on the pRG/III-hs-MBP plasmid (Tucker and Grisshammer 1996). The different sequence parts are labeled for maltose-binding protein (MBP), glycine-serine linker (GS)_5_, HRV 3C protease cleavage sites (3C, residues LEVLFQ↓GP), NdeI and AgeI restriction sites, thioredoxin A (TrxA), and a deca-histidine tag (H_10_). **b**–**e** Expression and solubilization tests quantified by western blot. The number under each band represents its relative intensity compared to the strongest band in the gel. **b** Expression test using different *E. coli* strains. **c** Expression test in the absence (−) or in the presence (+) of 20 mg/L cholesterol. **d** Test of the influence of the expression duration after induction. **e** Test of membrane solubilization using different detergent mixtures. The solubilized fractions were subjected to nickel-affinity chromatography and eluted by the same buffer for all the samples (20 mM Tris pH 7.5, 350 mM NaCl, 250 mM imidazole, 0.15% DM) before quantification by western blot

The TS-β_1_AR mutant (Isogai et al. 2016) was first cloned from the insect cell vector into the *E. coli* expression vector pRG/III-hs-MBP (Tucker and Grisshammer 1996). In the original pRG/III-hs-MBP, TS-β_1_AR is linked to the N- and C-terminal MBP and Trx fusion proteins via thrombin protease cleavage sites. However, cleavage by thrombin resulted in aggregation of the receptor. Therefore, we replaced these sites by cleavage sites for the more selective HRV 3C protease (Waugh 2011), and additionally separated them on each side from the protein sequences by (GS)_5_ linkers (Fig. 2a). Using HRV 3C overcame the aggregation problem, while the (GS)_5_ linkers increased the efficiency of HRV 3C cleavage by about 70%, apparently by making the cleavage sites more accessible. To facilitate the use of the construct for other GPCRs or transmembrane proteins and replacement by respective genes of interest, we also added the two endonuclease restriction site sequences NdeI and AgeI upstream and downstream of the β_1_AR gene (Fig. 2a). The YY-β_1_AR mutant vector, which encodes the G protein binding-competent β_1_AR, was obtained from this TS-β_1_AR vector by reintroducing the two native tyrosines via the point mutations A227Y and L343Y.

### Protein expression

Following previous experience (Supplementary Table S1), the β_1_AR expression in *E. coli* was carried out at a reduced temperature of 22 °C as described previously to slow down protein synthesis and increase the efficiency of lipid bilayer insertion (Hampe et al. 2000; Weiß and Grisshammer 2002; White et al. 2004; Shibata et al. 2009). Minimal media were used throughout to optimize for isotope labelling in NMR experiments. Several parameters were screened to improve the expression levels of the TS-β_1_AR vector as quantified by western blot against the C-terminal His_10_ tag. Of the four tested *E. coli* strains standard BL21(DE3) and its derivatives pLysS, Rosetta 2, and (CodonPlus) RIL (Fig. 2b), Rosetta 2 achieved the highest expression level. This was followed by 20–30% lower expression levels for pLysS and RIL. The standard BL21(DE3) cells expressed only a third of Rosetta 2. The higher expression levels of Rosetta 2 and RIL must result from their additional tRNAs for rare *E. coli* codons. Of note, the TS-β_1_AR gene had been taken from the insect cell expression vector and not been codon-optimized for *E. coli*. As an alternative, the receptor gene may be codon-optimized for *E. coli,* but such a strategy may become costly when studying a large number of different receptors (Petrovskaya et al. 2010). The increased expression level of pLysS may be attributed to the better suppression of basal expression in these cells.

We also tested the effect of cholesterol, since it has been reported to stabilize GPCRs (Gimpl and Fahrenholz 2002; Pucadyil and Chattopadhyay 2006; Prasanna et al. 2014) and indeed also stabilizes TS-β_1_AR (see below). However, the addition of 20 mg/L cholesterol to the growth medium had no effect on the expression level (Fig. 2c), but retarded considerably (~ twofold) *E. coli* growth. Also a longer expression time (48 h) showed no improvement in comparison to overnight cultures (Fig. 2d).

Based on these test results, all subsequent expressions were carried out using Rosetta 2 without addition of cholesterol in overnight cultures.

### Membrane solubilization

Three different conditions were tested for membrane solubilization: 1.5% DM, 1.5% DM + 0.7% CHAPS + 0.15% CHS (cholesteryl hemisuccinate), and 1.5% DDM + 0.7% CHAPS + 0.15% CHS (Fig. 2e). We had previously used 1.5% DM to extract β_1_AR from the insect cell membranes with good results. However, the DM extraction efficiency was very low for the *E. coli* membrane and increased considerably when 0.7% CHAPS + 0.15% CHS were added. The efficiency further increased when replacing DM by the longer-chain DDM. Thus the most widely used detergent mixture to extract GPCRs from *E. coli* membranes (Fig. 1b) also proved most efficient for TS-β_1_AR. Of note, addition of CHS also increases the TS-β_1_AR temperature stability (see below).

As we had previously found that the quality of NMR spectra and long-term stability was best for TS-β_1_AR in DM micelles (Isogai et al. 2016), the β_1_AR mutants solubilized by the DDM/CHAPS/CHS mixture from the *E. coli* membrane were exchanged to DM during the subsequent purification steps, which were carried out with 0.15% (nickel affinity) or 0.1% of DM (ion exchange and alprenolol affinity).

### Purification strategy

**Fig. 3:**
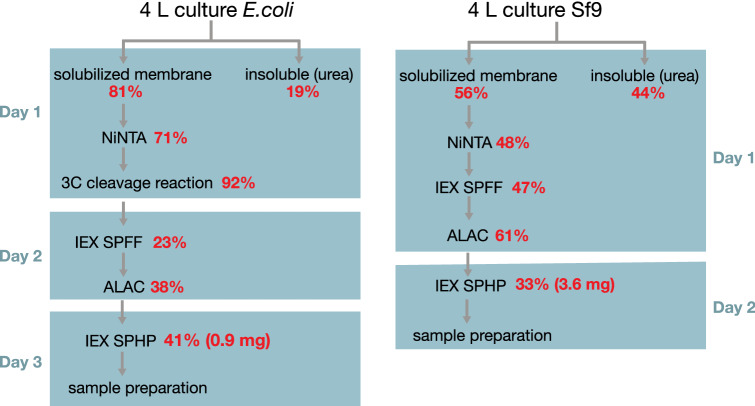
Purification schemes with timeline and yields for β_1_AR expressed in *E. coli* and Sf9 cells. Each work day of the purification is indicated by a blue box. Receptor yields estimated by quantifying the SDS or western-blot gels are indicated relative to the previous step by red numbers

The purification procedure after the membrane solubilization was adapted from the previous protocol for insect cell-expressed β_1_AR (Isogai et al. 2016). The individual steps of both *E. coli* and insect cell purification protocols are shown in Fig. 3. The purification consists sequentially of nickel affinity, fast-flow cationic ion exchange, alprenolol affinity (ALAC), and high-performance cationic ion exchange chromatography steps. This is based on the following rationale. The first, fast flow cation exchange (SPFF) chromatography removes many impurities, which are still present in the nickel-eluted fraction, and concentrates the receptor from the large elution volume (90 mL to ~ 25 mL for 4 L culture). This is important for the subsequent ALAC step, since the alprenolol-sepharose resin has low binding capacity (Caron et al. 1979) and the ALAC column requires slow loading (0.1 mL/min). The ALAC step retains only the ligand binding-competent protein and is crucial for a homogeneous sample. The second, high-performance cation exchange (SPHP) chromatography following the ALAC confers an ultra-pure sample and also concentrates the receptor in a mild way without aggregation from the 60-mL ALAC eluate to about 4 mL.

The major difference of the *E. coli* β_1_AR construct relative to the insect cell construct is the presence of the MBP and TrxA fusion partners. Thus it is a crucial question at which point of the protocol these fusion partners are removed by cleavage. Since MBP, TS-β_1_AR and TrxA have very distinct isoelectric points (4.8, 9.6, and 4.3, respectively), the fusion protein is hard to purify by either cationic or anionic ion exchange chromatography. In contrast, the cleaved β_1_AR is readily separated from MBP and TrxA by ion exchange chromatography. Indeed, cleavage carried out directly after the nickel affinity step instead of the ALAC increased the yield of purified TS-β_1_AR from 0.17 to 0.25 mg/L in a direct comparison.

### Quantitative analysis of the purification steps

The efficiencies of the individual steps in the *E. coli* and insect cell purification protocols were determined by quantifying western blots or Coomassie-stained gels (Supplementary Figure S1). These efficiencies are indicated in the overall flow diagrams for the two protocols (Fig. 3). Of note, the *E. coli* purification extends over three days instead of the two days for the insect cell purification due to the additional overnight cleavage step.

Apparently, solubilization was more efficient from *E. coli* (81%) than from insect cells (56%). As this might have been caused by the different detergent used (1.5% DDM, 0.7% CHAPS, 0.15% CHS vs. DM), we also tested solubilization of the insect cell material by the DDM/CHAPS/CHS mixture. However, no increase in efficiency was observed. Thus the effect must be due to the different membrane composition of *E. coli* and insect cells. During the following nickel affinity step, the detergent is exchanged for the *E. coli* material to DM. During this step considerably more β_1_AR was recovered from the *E. coli* material (71%) than from the insect cell material (48%). A test with DDM/CHAPS/CHS-solubilized insect cell material showed that the higher yield for the *E. coli* material in this step was not due to the different detergent mixture. The following cleavage of the *E. coli* material by the HRV 3C protease was highly efficient (92%). After this step both *E. coli* and insect cell-derived β_1_AR samples should be identical in their composition.

Considerable amounts of material are lost in the subsequent purification steps. From the β_1_AR *E. coli* material only 23% are recovered after the SPFF ion exchange step in contrast to 47% for the insect cell material. Similarly, also the ALAC column is less efficient for the *E. coli* (38%) vs. the insect cell (61%) material. We attribute these higher losses for the *E. coli* material to a appreciably larger fraction of misfolded protein present in this preparation. After filtering out these misfolded receptors, the second cationic exchange elution has similar efficiency for both preparations (41% *E. coli* vs. 33% insect cell).

The final yields for TS-β_1_AR and the less stable YY-β_1_AR mutant purified from *E. coli* were 1.2 and 0.9 mg from 4-L minimal media cultures, respectively. These yields are about 4 times smaller than from insect cell cultures, but similar to other GPCRs produced from *E. coli* under native conditions, such as the CB1 and α_1A_ receptors (Link et al. 2008; Wu et al. 2020).

### Thermal stability quality tests

**Fig. 4:**
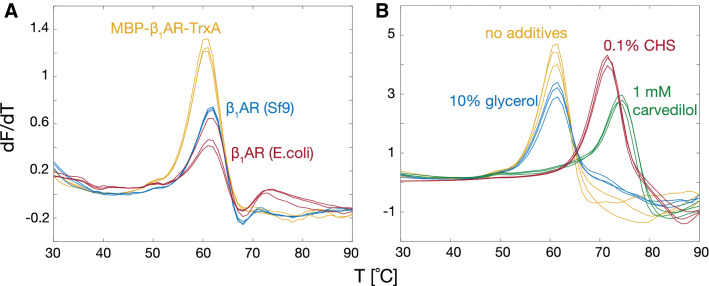
Quality test of *E. coli*-derived β_1_AR by the CPM thermal shift assay. **a** Thermal stability of *E. coli*-derived full-length MBP-TS-β_1_AR-TrxA and TS-β_1_AR in comparison to insect-derived TS-β_1_AR (all apo forms). Samples contained ~ 25 μM receptor in 20 mM Tris, 1 mM EDTA, 750 mM NaCl, 0.1% DM, pH 7.5. **b** Thermal stability of *E. coli*-derived TS-β_1_AR in the presence of various additives. The CPM fluorescence signal (F) is shown as its first derivative (dF/dT). Triplicates of the experiments are shown color-coded by identical colors

As a first test of the quality of the *E. coli*-derived receptors we used the common CPM (N-[4-(7-diethylamino-4-methyl-3-coumarinyl)phenyl] maleimide) microscale fluorescent stability assay. Both *E. coli*- and insect cell-derived apo TS-β_1_AR showed the same well-defined melting temperature T_m_ of 62 °C (Fig. 4a). Of note, the melting temperature of the entire, uncleaved MBP-TS-β_1_AR-TrxA fusion protein (T_m_ = 61 °C) is also close to the values of the isolated receptor. As observed previously for insect cell-derived β_1_AR (Warne et al. 2009), binding of the antagonist carvedilol increased T_m_ for the *E. coli*-derived TS-β_1_AR by about 15 °C (Fig. 4b). Also the addition of 0.1% CHS increased T_m_ to 72 °C, while glycerol showed no effect.

### Isotope labeling and NMR spectra

**Fig. 5:**
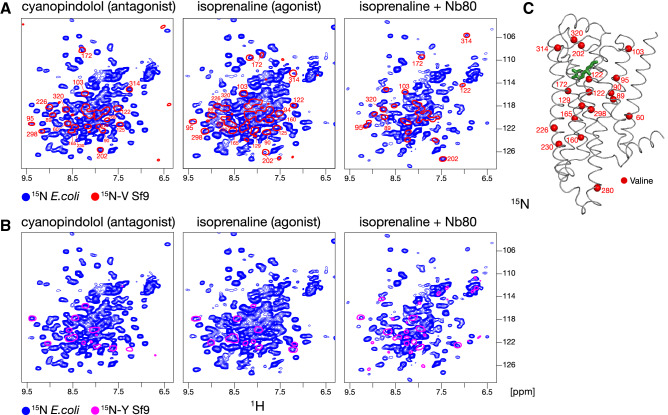
Comparison of ^1^H-^15^N TROSY spectra of ^15^N-labeled YY-β_1_AR derived from *E. coli* and Sf9 insect cells. Spectra are shown for YY-β_1_AR binary complexes with the antagonist cyanopindolol and the agonist isoprenaline, as well as the ternary complex with isoprenaline and the G protein-mimicking Nb80. All the experiments were carried out at 304 K, with samples containing ~ 100 μM receptor complexes in 20 mM Tris, 1 mM EDTA, 100 mM NaCl, 37 mM DM, 0.02% NaN_3_, 5% D_2_O, pH 7.5. **a** Superposition of spectra of uniformly ^15^ N-labeled YY-β_1_AR obtained from *E. coli* (blue) and ^15^ N-valine labeled YY-β_1_AR obtained from Sf9 cells (red). **b** Superposition of spectra of uniformly ^15^N-labeled YY-β_1_AR obtained from *E. coli* (blue) and ^15^N-tyrosine labeled YY-β_1_AR obtained from Sf9 cells (magenta). Where available, resonances are marked with assignment information. **c** Crystal structure of β_1_AR in complex with isoprenaline (green, PDB code 2Y03). Valine residues observed and assigned in panels **a** and **b** are shown as red spheres

^15^N-labeled samples of TS-β_1_AR and YY-β_1_AR were produced with no difficulties using the common *E. coli* M9 minimal medium for isotope labelling (Green et al. 2012). As a test for proper function, ^1^H-^15^N TROSY spectra of ^15^N-YY-β_1_AR (Fig. 5) were recorded in binary complexes with the antagonist cyanopindolol, the agonist isoprenaline, and in the ternary complex with isoprenaline and the nanobody Nb80, which binds in the same way to the intracellular side of the receptor as the full G protein (Rasmussen et al. 2011). For each of these distinct functional states, well-dispersed ^1^H-^15^N resonances were observed, which are typical of a properly folded protein. Figure 5 shows a superposition of these spectra from uniformly ^15^N-labeled YY-β_1_AR obtained from *E. coli* with spectra from selectively ^15^N-valine (panel A) or ^15^N-tyrosine (panel B) labeled YY-β_1_AR obtained from insect cells in complexes with the same ligands. Clearly the resonances from the sparsely labeled insect cell samples are in very similar positions. In particular, the ^1^H-^15^N resonances of V172, V202 and V314, which are located close to the orthosteric ligand head group and the extracellular entrance of the ligand pocket (Fig. 5c), show identical strong variations in the various complexes. These have been identified previously (Abiko et al. 2019; Grahl et al. 2020) with the inactive conformation (cyanopindolol), a mixture in slow chemical exchange between preactive and active conformation (isoprenaline), and the active conformation (isoprenaline + Nb80).

For a more direct comparison with the selectively ^15^N-valine labeled receptor (TS-β_1_AR) obtained from insect cells, we also attempted ^15^N-valine selective labeling in *E. coli* by the addition of ^15^N-valine to the unlabeled M9 medium. This yielded the same amount of protein as from uniformly ^15^N-labeled M9. The TROSY spectrum of cyanopindolol-bound TS-β_1_AR shows similar resonances as the spectrum of ^15^N-valine labeled TS-β_1_AR from insect cells, e.g. for V172, V202, and V314 (Supplementary Figure S2). However, due to the metabolic scrambling of ^15^N-valine to alanine, isoleucine, glutamate and other branched amino acids (Lacabanne et al. 2018; Sugiki et al. 2018), many additional non-valine resonances are observed for the *E. coli*-derived receptor whereas the valine resonances are relatively weakened (e.g. V95, which is already weak in the insect cell spectrum, is not even detected in the *E. coli* spectrum). We attribute these effects to the depletion of the ^15^N-labeled valine pool from the efficient two-way scrambling and also to the higher mobility of non-valine residues located in surface loops or tails. In principle, the scrambling may be reduced by the use of auxotrophic *E. coli* strains (Lin et al. 2011), but this approach was not further pursued.

We also tested the production of uniformly ^2^H,^15^N-labeled TS-β_1_AR in *E.coli* by growing the bacteria in ^15^N-labeled M9 medium and 100% D_2_O using standard protocols (Opitz et al. 2019). Protein expression was about half of the amount obtained in ^15^N M9 medium in H_2_O. Such reductions in yield are not uncommon for deuterated media, since bacterial growth and metabolism are affected by deuteration (Opitz et al. 2019). Improvements in yield may be achievable by multiple rounds of adaptation and selection or the use of optimized strains (Paliy et al. 2003; Kelpšas and Wachenfeldt 2019).

**Fig. 6:**
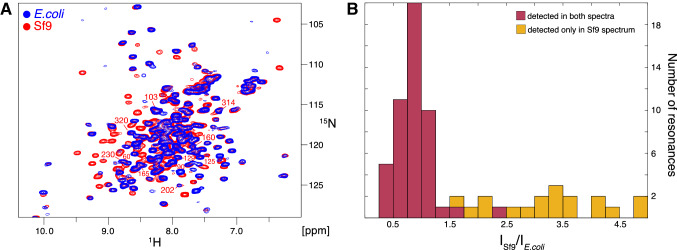
Comparison of ^1^H-^15^N TROSY spectra of ^2^H,^15^N-labeled YY-β_1_AR derived from *E. coli* and Sf9 insect cells. **a** ^1^H-^15^N TROSY spectra of 17 μM uniformly ^2^H,^15^N-labeled TS-β_1_AR derived from *E. coli* (blue) and of 90 μM 60% ^2^H, 90% ^15^N-labeled TS-β_1_AR expressed in baculovirus-infected Sf9 insect cells (red). Both samples contained 1 mM of the antagonist alprenolol in 20 mM Tris, 1 mM EDTA, 100 mM NaCl, 37 mM DM, 0.02% NaN_3_, 5% D_2_O, pH 7.5. Experiments were carried out at 304 K with total experimental times of 67 h and 10.5 h for the *E. coli* and Sf9 samples, respectively. **b** Histogram of intensity ratios (I_Sf9_/I_*E. coli*_) of Sf9 and corresponding *E. coli* ^1^H-^15^N TROSY resonances. Intensities ratios of resonances present in both spectra are shown as brown histogram bars. For resonances, which were only detected in the Sf9 sample, I_Sf9_/I_*E. coli*_ was approximated by the intensity of the Sf9 resonance divided by 3 times the standard deviation of the noise of the *E. coli* spectrum. These cases then present a lower limit for I_Sf9_/I_*E. coli*_ and are shown in dark yellow

An NMR sample of ^2^H,^15^N-labeled TS-β_1_AR in complex with the antagonist alprenolol was prepared from this *E. coli* culture by carrying out the entire solubilization and purification procedure in H_2_O. Figure 6a shows a comparison of TROSY spectra of this ^2^H,^15^N-labeled TS-β_1_AR alprenolol complex and an identical complex prepared from insect cell-derived TS-β_1_AR with about 60% ^2^H and 90% ^15^N incorporation. The latter was obtained by growing insect cells in H_2_O and supplying ^2^H/^15^N-labeled amino acids via respectively labeled yeast extract (Opitz et al. 2015). Clearly the ^1^H/^15^N resonance positions are very close in both spectra indicating that the ^2^H,^15^N-labeled TS-β_1_AR from *E. coli* is in the same well-folded state as the TS-β_1_AR from insect cells. However, fewer ^1^H/^15^N resonances are observed for the *E. coli* material. A histogram of the intensity ratios of the resonances from the insect cell and *E. coli* samples (Fig. 6b) reveals that about a third of the resonances are significantly more intense in the insect cell sample. We attribute this effect to the incomplete back exchange of amide protons during the purification in H_2_O. This is corroborated by the observation that most of the missing resonances are located in the ^1^H^N^ downfield region. These ^1^H^N^ nuclei are part of short hydrogen bonds in well-formed, stable secondary structure elements (Grzesiek et al. 2004). It is obvious that better back exchange of amide protons is necessary for the *E. coli*-derived ^2^H,^15^N-labeled TS-β_1_AR. This may achievable by slight destabilization of the receptor at higher temperature, higher pressure or by chemical denaturants. Other researcher in the field have reported similar problems of amide proton back exchange for GPCRs grown in 100% deuterated media (K. Wüthrich, personal communication).

## Conclusions

We have presented here an optimized protocol for *E. coli* expression and purification of two β_1_AR constructs*,* which had previously been engineered in insect cells for thermal stability and subsequent successful structural and dynamical studies. Of note, this protein engineering comprises the removal of the N-terminal glycosylation and C-terminal palmitoylation, as is often the case in solved GPCR structures (Munk et al. 2019). Thus the absence of the respective PTM machineries did not present a problem for the *E. coli* expression. Our protocol uses the same genes as in the insect cells and was optimized for 4-L cultures, yielding 0.9 mg of the fully active YY-β_1_AR, and 1.2 mg for the more thermostable TS-β_1_AR construct, which is about a quarter of the insect cell yield.

The *E. coli* sample quality is identical to the insect cell material as assayed by the thermal melting behavior and a direct comparison of backbone ^1^H-^15^N spectra of the same receptor constructs obtained from insect cells in 3 different functional states. As such the *E. coli*-derived β_1_AR is usable for any structural or biophysical study in the same way as β_1_AR derived from insect cells.

While the purification steps and time are similar for insect cell- and *E. coli*-derived receptors, genetic manipulations are much simpler and protein expression is much faster in *E. coli*. The production of baculovirus and the scaling-up of insect cell cultures typically takes weeks, whereas bacterial expression can be achieved within two days. Furthermore, *E. coli* offers the advantage of efficient, low-cost, and highly developed procedures (Boisbouvier and Kay 2018) for uniform and very specific isotope labeling.

The approach presented here should be easily transferable to other GPCRs of interest by using the standard restriction sites introduced into the expression plasmid and by comparing the yields at the described quantitative checkpoints in the purification procedure.

## Materials and methods

### Generation of expression constructs

An optimized expression construct for the turkey β_1_AR receptor was derived from the vector pRG/III-hs-MBP kindly provided by R. Grisshammer (NIH) by (i) replacement of the NTS1 gene by the thermostabilized TS-β_1_AR receptor DNA sequence by restriction-free cloning (van den Ent and Löwe 2006), (ii) introduction of two 3C cleavage sites between the MBP and TS-β_1_AR and between the TS-β_1_AR and TrxA sequences by site-directed mutagenesis, (iii) addition of two (GS)_5_ spacers before and after each 3C cleavage site by megaprimer cloning (Zhang et al. 2017), and (iv) introduction of NdeI and AgeI cleavage sites upstream and downstream of the TS-β_1_AR gene sequence by site-directed mutagenesis (Hemsley et al. 1989). The final fusion protein sequence is summarized in Fig. 2a and the TS-β_1_AR sequence after the cleavage of fusion proteins by 3C protease is the following:

- GPGSGSGSGSGHMGAELLSQQWEAGMSLLMALVVLLIVAGNVLVIAAIGSTQRLQTLTNLFITSLACADLVVGLLVVPFGATLVVRGTWLWGSFLCELWTSLDVLCVTASVETLCVIAIDRYLAITSPFRYQSLMTRARAKVIICTVWAISALVSFLPIMMHWWRDEDPQALKCYQDPGCCEFVTNRAYAIASSIISFYIPLLIMIFVALRVYREAKEQIRKIDRASKRKTSRVMLMREHKALKTLGIIMGVFTLCWLPFFLVNIVNVFNRDLVPKWLFVAFNWLGYANSAMNPIILCRSPDFRKAFKRLLAFPRKADRRLLEGTGSGSGSGSLEVLFQ

For the YY-β_1_AR construct, the two native tyrosine residues Y227 and Y343 were introduced into TS-β_1_AR sequence by site-directed mutagenesis. The molecular weight of both TS-β_1_AR and YY-β_1_AR proteins is ~ 38 kDa.

### *Expression of uniformly *^*15*^*N-labeled β*_*1*_*AR in E. coli*

Chemically competent Rosetta 2(DE3) (Novagen) cells were transformed with the optimized expression vectors by heat shock and frozen as a glycerol stock. 100 μL thawed glycerol stock were then used to grow a 5-mL inoculum in Luria–Bertani (LB) medium at 37 °C for 9–10 h. The LB culture was diluted with ~ 100 mL M9 minimal medium (supplemented with 4 g/L of glucose and 1 g/L of ^15^NH_4_Cl) to OD_600_ = 0.1 and grown overnight at 37 °C. On the next day, the culture was diluted into 4 L M9 to OD_600_ = 0.1 and grown at 37 °C until the OD_600_ reached 0.6. Subsequently, the temperature was reduced to 22 °C and the culture further grown to OD_600_ = 0.9. At this point, the expression was induced with 0.25 mM isopropyl-β-D-thiogalactoside (Sigma-Aldrich) and growth continued for 16 h at the same temperature. Then cells were pelleted for 15 min at 5000 r.p.m., resuspended in 70 mL 20 mM Tris pH 7.5, 1 mM EDTA containing 2 tablets of cOmplete™ protease inhibitor cocktail (Hoffmann-La Roche), flash-frozen in liquid nitrogen, and stored at -80 °C until purification.

For testing the effect of cholesterol on expression, a cholesterol stock solution of 20 g/L was prepared in ethanol and added to the M9 media to a final concentration of 20 mg/L before inoculation.

### *Expression of *^*15*^*N-valine-labeled β*_*1*_*AR in E. coli*

^15^N-valine-labeled β_1_AR in *E. coli* was expressed as described for the ^15^N-labeled protein with the exception of adding 100 mg/L ^15^N-valine (Sigma-Aldrich) and 30 mg/L leucine to the ^14^N_4_HCl-containing M9 medium.

### *Expression of uniformly *^*2*^*H,*^*15*^*N-labeled TS-β*_*1*_*AR in E. coli*

Uniformly ^2^H,^15^N-labeled TS-β_1_AR was expressed in *E. coli* using M9 medium prepared in D_2_O (> 90%) supplemented with 4 g/L of glucose and 1 g/L of ^15^NH_4_Cl as described for protonated M9. Prior to expression, cells were gradually adapted to the deuterated medium by 9–16 h steps of growth at 37 °C in 0/100, 30/70, 70/30, and 100/0% deuterated/protonated medium, respectively.

### Membrane fraction preparation

Unless otherwise stated, all β_1_AR purification steps were carried out at 4 °C. Thawed cells from a 4-L culture were passed 3 times through a pre-cooled French pressure cell at 2000 psi and ultra-centrifuged at 140,000×*g* for 1 h. For solubilization, the pellet was suspended in 120 mL 20 mM Tris pH 7.5, 400 mM NaCl, 2.5 mM imidazole, 1.5% DM, 0.7% CHAPS, 0.15% CHS (all detergents obtained from Anatrace). Solubilization was achieved in three steps: (1) 2 × 5 s treatment with an electric homogenizer (Ultra TURRAX™, IKA) equipped with a 7-mm saw tooth dispersing probe at 11.000 r.p.m.; (2) homogenization with a tight-piston Dounce homogenizer; and (3) 2 h of incubation under gentle motion in a roller mixer (Stuart Scientific). The solubilized cells were then ultra-centrifuged at 140,000×*g* for 30 min. The supernatant containing the solubilized receptor was used for further purification, whereas ¼ of the pellet was resolubilized in 30 mL 20 mM Tris pH 7.5, 200 mM NaCl, 7 M urea for quantification of the remaining insoluble receptor.

### Membrane solubilization test

For quantifying the efficiency of membrane solubilization by different detergents, the cells from a 2-L *E. coli* culture were disrupted, divided into 3 equal parts, and centrifuged as described above. The 3 pellets were then suspended, each in 10 mL 20 mM Tris pH 7.5, 400 mM NaCl, 2.5 mM imidazole + either 1.5% DM or (1.5% DM + 0.7% CHAPS + 0.15% CHS) or (1.5% DDM + 0.7% CHAPS + 0.15% CHS). Solubilization under mechanical agitation and further centrifugation were then carried out as described above. The solubilized supernatant of each sample was purified by Ni affinity and quantified for receptor content by western blot as described below.

### Protein purification

The solubilized membrane fraction was incubated for 2 h in a rotator mixer with 15 mL (50% slurry) of Ni–NTA agarose resin (Qiagen) which had been pre-equilibrated with 20 mM Tris pH 7.5, 350 mM NaCl. The resin was washed using two centrifugation (5 min, 1000 r.p.m, in an Eppendorf 5430 R centrifuge) and resuspension steps (90 mL 20 mM Tris pH 7.5, 850 mM NaCl, 15 mM imidazole, 0.15% DM; 70 mL of 20 mM Tris pH 7.5, 350 mM NaCl, 15 mM imidazole, 0.15% DM). The washed resin was placed in a column on top of 2 mL additional resin. β_1_AR was eluted by gravity flow with 90 mL 20 mM Tris pH 7.5, 350 mM NaCl, 250 mM imidazole, 0.15% DM. This eluted fraction was then incubated overnight with 20 mg of home-made 3C protease [produced according to (Ullah et al. 2016)] in a rotator mixer (Stuart Scientific).

On the following day, this fraction incubated with 3C protease was diluted to a volume of 180 mL with buffer IEX A (20 mM Tris pH 7.5, 1 mM EDTA, 0.1% DM) and applied to a 5 mL SP Sepharose™ Fast Flow cation exchange column (GE healthcare), washed with 20 mL of buffer IEX A, and eluted with a gradient from 5 to 100% of buffer IEX B (20 mM Tris pH 7.5, 1 mM EDTA, 2 M NaCl, 0.1% DM). β_1_AR eluted at ~ 30% of IEX B. The eluted peak was collected and applied to a 5-mL alprenolol-sepharose affinity column (ALAC) prepared as described in (Hemsley et al. 1989). Non-binding protein was slowly washed out (0.1 mL/min) with 60 mL 20 mM Tris pH 7.5, 1 mM EDTA, 750 mM NaCl, 0.1% DM and the binding-competent β_1_AR was eluted with 60 mL of the same buffer containing 1 mM of the inverse-agonist atenolol.

The eluted fraction from the ALAC was diluted to 200 mL with buffer IEX A and applied to a 5-mL SP Sepharose™ High Performance column (GE healthcare), washed with 20 mL buffer IEX A, and eluted with a gradient from 7 to 90% of IEX B. The pure, homogeneous β_1_AR eluted at ~ 49% of IEX B. This fraction was collected, buffer-exchanged to 20 mM Tris pH 7.5, 1 mM EDTA, 100 mM NaCl, 0.1% DM and concentrated to ~ 250 μL using a 50-kDa molecular weight cut-off Amicon centrifugal filter (Millipore). Receptor concentrations were estimated by OD_280_ using a Nano-Drop (Thermo Scientific™) UV–Vis spectrophotometer and a theoretical extinction coefficient of 66,055 M^−1^ cm^−1^.

For complex formation with the higher-affinity, ortho-steric ligands cyanopindolol or isoprenaline, the low-affinity ligand alprenolol was exchanged as described previously (Isogai et al. 2016). In brief, the samples were washed repeatedly first with buffer devoid of ligand and then with buffer containing the higher-affinity ligand of interest. Final ligand concentrations were 1 mM cyanopindolol or 2 mM isoprenaline (+ 20 mM sodium ascorbate for stability). For formation of the ternary YY-β_1_AR complex, a 1.2 molar equivalent of Nb80 [prepared as described previously (Rasmussen et al. 2011)] was added to the preformed isoprenaline∙YY-β_1_AR complex.

### *Expression and purification of selectively *^*15*^*N-valine-, *^*15*^*N-tyrosine-, and uniformly *^*2*^*H,*^*15*^*N-labeled β*_*1*_*AR from Sf9 insect cells*

Expression of the ^15^N-valine-labeled YY-β_1_AR in baculo-virus-infected Sf9 cells, purification, and assignments were carried out as described previously (Isogai et al. 2016). ^15^N-tyrosine labeled YY-β_1_AR was expressed similar to the^15^N-valine-labeled receptor by replacing the 100 mg/L ^15^N-valine by 150 mg/L ^15^N-tyrosine in the expression media. 60% ^2^H, 90% ^15^N-labeled TS-β_1_AR was expressed in baculovirus-infected Sf9 insect cells grown on ^2^H-^15^N-labeled yeast extract and purified as described previously (Opitz et al. 2015).

### Quantitative analysis of receptor from gel electrophoresis and western blotting

At every step of the purification, the amount of receptor was quantified by SDS/PAGE followed by western blotting against the histidine-tagged receptor using chemiluminescence. For this, 10 μL of the respective fractions were diluted with 10 μL SDS–PAGE buffer (125 mM Tris–HCl pH 6.8, 4% (w/v) SDS, 20% (v/v) glycerol and 0.01% (w/v) bromophenol blue) and loaded onto Mini-PROTEAN TGX™ 4–20% precast gels (Bio-Rad). As a reference, 7 μL of Precision Plus Protein™ dual color standard weight ladder (#1610374, Bio-Rad) were also loaded onto the gel, and the proteins separated for 30 min at 200 V. The gel was then stained with Coomassie brilliant blue and images recorded using an Epson Perfection V800 Photo scanner at 600 dpi resolution. Fractions from the different steps of the preparation loaded onto the same gel were quantified in their relative β_1_AR amount by densitometry (see below).

For better identification and quantification β_1_AR was also analyzed by western blots in the early purification steps. For this, proteins were transferred to 0.2 μm nitrocellulose membranes using the Trans-Blot Turbo™ transfer system (Bio-Rad). Western-blot membranes were treated with HRP anti-His tag antibody (BioLegend) and Lumi-Light Western Blotting Substrate (Hoffmann-La Roche). The chemiluminescence signal was detected using a Fusion FX6 reader (Vilber) at 600 dpi resolution.

Individually selected bands of the obtained images from the SDS-PAGE gels and from the western blot membranes were quantified by ImageJ (Schindelin et al. 2012) after background subtraction using the rolling ball radius method (50 pixels).

### Thermal stability assays

The melting temperatures of the full-length MBP-TS-β_1_AR-TrxA fusion and of TS-β_1_AR isolated from *E. coli* as well as insect cells under various conditions were determined by following the fluorescence of added *N*-[4-(7-diethylamino-4-methyl-3-coumarinyl)phenyl]maleimide (CPM) using a Qiagen Rotorgene QPCR thermocycler. For this, ~ 1.5 μL of ~ 1 mg/mL receptor were diluted in 20 mM Tris pH 7.5, 1 mM EDTA, 750 mM NaCl, 0.1% DM, 1 mM atenolol (and optionally 10% glycerol, 0.1% CHS, or 1 mM carvedilol) to a final volume of 60 μL and incubated for 1 h on ice. After incubation, 5 μL of a 186 μM solution of CPM in DMSO was added to each sample (final concentration ~ 14 μM). The GPCR thiol-CPM conjugate fluorescence (excitation 387 nm, emission 463 nm) was monitored while increasing the temperature from 25 to 90 °C at a rate of 2 °C/minute. For every condition, 3 replicates were measured.

### NMR experiments

NMR samples were prepared in 5-mm Shigemi tubes as ~ 250-μL volumes of ~ 100 μM protonated (~ 20 μM *E. coli*-derived deuterated) receptor complexes in 20 mM Tris pH 7.5, 1 mM EDTA, 100 mM NaCl, 37 mM DM, 0.02% NaN_3_, 5% D_2_O. All NMR experiments were carried out on a Bruker AVANCE 21.2 T (900 MHz) spectrometer equipped with a TCI cryoprobe at a temperature of 304 K. Unless stated otherwise, ^1^H-^15^N TROSY experiments were recorded as 80/120 (^15^N) × 1024 (^1^H) complex points with acquisition times of 16/24 ms (^15^N) and 42 ms (^1^H) for protonated/deuterated samples, respectively. For optimal sensitivity, the ^1^H-^15^ N transfer time was reduced to 3.0 ms using a 1 s interscan delay. Typical total experimental times were 48 h.

All NMR spectra were processed with NMRPipe (Delaglio et al. 1995) and evaluated with CcpNmr Analysis (Vranken et al. 2005).

## Supporting information

Supplementary Figures S1, S2 showing details of β_1_AR expression optimization and purification as well as TROSY spectra of ^15^N-valine selectively labeled TS-β_1_AR; Supplementary Table S1 giving an overview of the most important approaches used in previous studies of GPCR expression in *E. coli*.

## Supplementary Information

Below is the link to the electronic supplementary material.

Electronic supplementary material 1 (XLSX 26 kb)

Electronic supplementary material 2 (PDF 1635 kb)

## Data Availability

All data generated or analyzed during this study are included in this published article and its supplementary information files.
