# Supplementary material for: Efficient production of a functional G protein-coupled receptor in E. coli for structural studies
Source: J Biomol NMR. 2021 Jan 27;75(1):25–38. doi: 10.1007/s10858-020-00354-6 (PMC7897205; doi:10.1007/s10858-020-00354-6)
Supplement: Supplementary file 2 — Electronic supplementary material 2 (PDF 1635 kb) [file 10858_2020_354_MOESM2_ESM.pdf]

Efficient production of functional β_1-adrenergic receptor in *E. coli* for structural studies

Layara Akemi Abiko*, Marco Rogowski, Antoine Gautier, Gebhard Schertler, and Stephan Grzesiek*

Focal Area Structural Biology and Biophysics, Biozentrum, University of Basel, CH-4056 Basel, Switzerland

Supporting Information

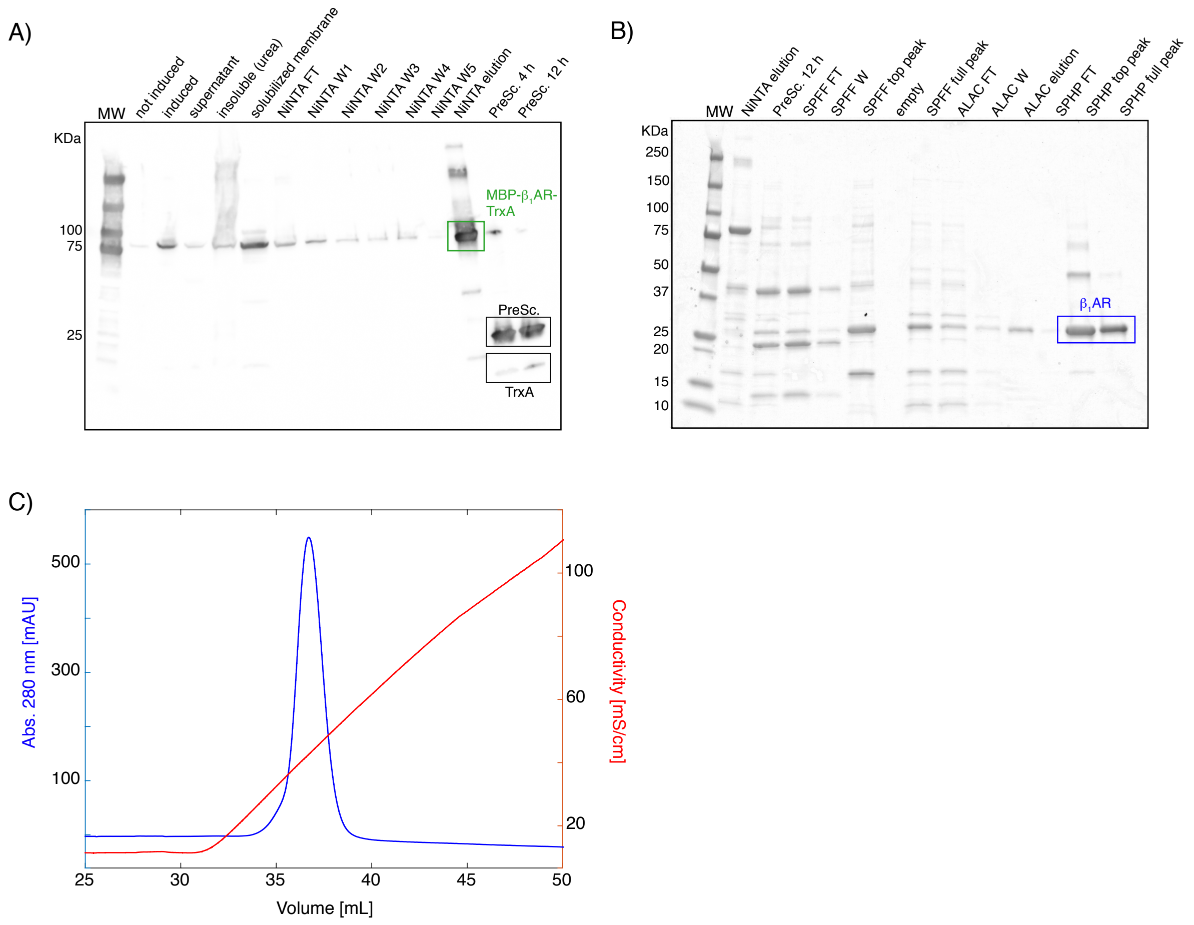

Figure S1. Quantification of TS-β_1AR *E. coli* expression and purification by gel electrophoresis and western blotting. Western blotting against the histidine tag was used for quantifying the early steps of membrane solubilization and purification (panel A), in which the TS-β_1AR band is not distinguishable due to the presence of other proteins. For the later steps (after nickel affinity chromatography), protein bands were quantified from an SDS-page gel (panel B). Details of the purification steps, sample preparation, and quantification using the program ImageJ are described in Materials and Methods. The labels indicate: “not induced”: *E.coli* cells before induction of protein expression, “induced”: *E.coli* cells after induction of protein expression by addition of IPTG, “supernatant”: supernatant of cell harvesting step, “insoluble urea”: insoluble fraction after membrane solubilization resuspended with buffer containing 7M urea, “solubilized membrane”: membrane fraction solubilized with detergents (DDM, CHAPS and CHS), “NiNTA FT”: flow through from nickel column, “NiNTA W1-W5”: wash fractions from nickel column, “PreSc, 4h and 12h”: Cleavage reaction of fusion protein with 3C PreScission protease after 4 and 12 h, respectively, “SPFF FT”: flow through from the fast flow cation exchange column (SPFF), “SPFF W”: wash fraction from SPFF column, “SPFF top peak”: center fraction of the absorption (280 nm) peak from the SPFF column, “empty”: empty lane, “SPFF full peak”: pool of all fractions of the TS-β_1AR peak in the SPFF, “ALAC FT”: flow through from the alprenolol

affinity column (ALAC), "ALAC W": wash fraction from ALAC column, "ALAC elution": pool of fractions within the peak corresponding to the TS-β_1AR in the ALAC, "SPHP FT": flow through from the high-performance cation exchange column (SPHP), "SPHP top peak": fraction with the higher absorption (280 nm) from SPHP column, "SPHP full peak": pool of fractions within the peak corresponding to the TS-β_1AR in the SPHP column.
The western blot of the 3C reaction sample (panel A) shows the 3C PreScission protease and the cleaved thioredoxin (identified in the figure), but not the maltose-binding protein or the TS-β_1AR bands because these two proteins do not carry a poly-histidine tag. The band of the fusion protein after the nickel affinity purification is marked by a green rectangle. The band for the TS-β_1AR monomer in the SDS-page gel (panel B) after the last purification step is marked by a blue rectangle. Very small amounts of higher TS-β_1AR oligomers are also visible in the concentrated 'top peak' fraction.
(C) Final purification step using high-performance cation exchange chromatography (SPHP). The elution profile of the 280 nm absorption of TS-β_1AR is shown in blue. The center of the TS-β_1AR peak elutes at a conductivity (red line) of about 40 mS/cm. SDS-page analyses of the 'full peak' and 'top peak' from the SPHP elution are shown in panel B.

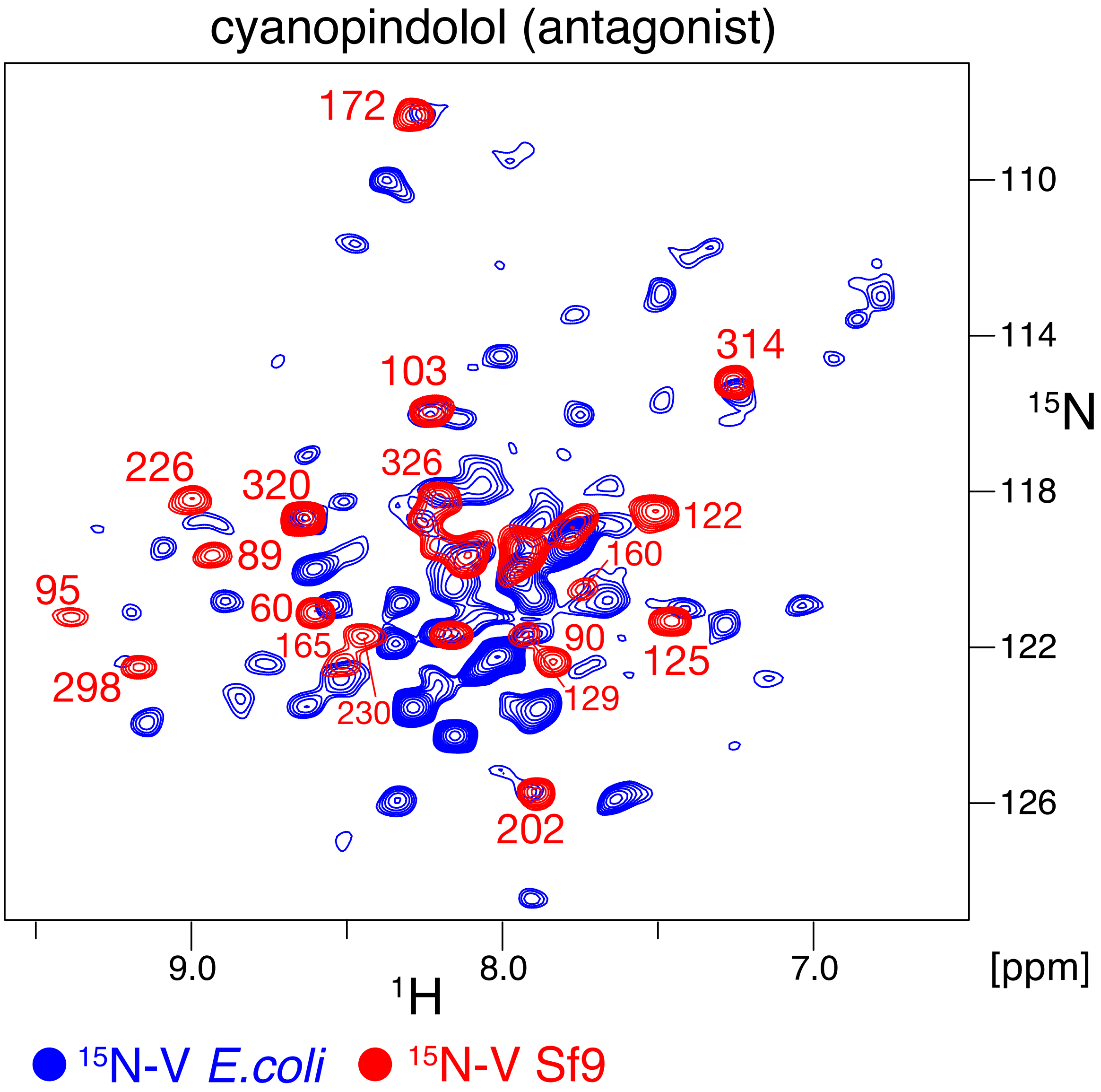

Figure S2. Comparison of ^{1}H-^{15}N TROSY spectra of ^{15}N-valine-labeled TS-β_1AR obtained from *E. coli* (blue) or insect cells (red) in complex with the antagonist cyanopindolol. Additional peaks and a relative weakening of valine resonances in the *E. coli* receptor spectra are the result of efficient metabolic scrambling in *E. coli* to alanine, isoleucine, leucine, glutamate and other branched amino acids. Both spectra were recorded for ~45 hours at 304 K on samples containing 165 µM (*E. coli*) or 150 µM (insect cell) TS-β_1AR, 1 mM cyanopindolol, 20 mM Tris, 1 mM EDTA, 100 mM NaCl, 37 mM DM, 0.02% NaN_3, 5% D_2O, pH 7.5.
